# Two Highly Immunized Hilly Areas *versus* Double Measles Outbreak Investigations in District Kangra, Himachal Pradesh, India, in 2006

**DOI:** 10.4103/0974-777X.52976

**Published:** 2009

**Authors:** Surender N Gupta, Naveen Gupta

**Affiliations:** *Regional Health and Family Welfare Training Centre (RHFWTC), Chheb, Kangra, Himachal Pradesh, India*

**Keywords:** Genotype, IgM antibodies, Measles, Outbreak, Polymerase chain reaction

## Abstract

**Background:**

We investigated two sequential outbreaks of measles in seven villages of Kangra, to confirm the diagnosis and to formulate recommendations for prevention and control.

**Methods:**

We defined a case of measles as occurrence of fever with rash in a child aged six months to 17 years during the period 3^rd^ September to 23^rd^ November 2006. We collected information on age, sex, residence, date of onset, symptoms, signs, treatment taken, traveling history and vaccination status. We described the outbreak by time, place and person. We estimated vaccine coverage and efficacy in the affected villages. We confirmed diagnosis clinically, serologically and through genotyping of the virus.

**Results:**

We identified 69 cases. Overall attack rates ranged between 4.2% and 6%. All case patients were between 6 years to 11 years of age. Age-specific attack rate in double outbreaks ranged in between 1.7% and 21.6%, the highest being in the age range 11–17 years. No deaths or complications were reported. The epidemic curve was suggestive of typical propagated pattern. The first outbreak imported virus after an interschool game competition (relative risk, 6.44%; 95% confidence interval, 3.81–10.91); followed by the second outbreak, in which people exchanged foods in the festival in one infected village of the first outbreak (relative risk, 5.3; 95% confidence interval, 1.90–14.77; *P* <.001). The calculated immunization coverage (93%) coincided nearly with administrative claims. The vaccine efficacies were estimated to be 85% and 81% in the first and second outbreaks respectively. Eleven of the 16 case patients were tested for measles IgM antibodies, while two nasopharyngeal swabs were positive by polymerase chain reaction (PCR) and are genotyped D4 measles strain. Vitamin A supplementations were only given in four villages.

**Conclusion:**

Measles outbreaks were confirmed in high–immunization-coverage areas. We recommended (i) second dose opportunity for measles in Himachal Pradesh and (ii) vitamin A supplementation to all the case patients.

## INTRODUCTION

Rubeola or measles, is the fifth leading cause of childhood mortality, especially in developing countries.[1] Failure to deliver at least one dose of measles vaccine to all infants remains the primary reason for high measles mortality and morbidity in developing countries like India and Pakistan and African countries.[2] But with the recommendation of second dose opportunity for measles by WHO/UNICEF in those districts achieving high routine vaccination coverage exceeding 90%, there was the largest percent reduction in estimated measles mortality during 1999-2005 in the western Pacific region (81%), followed by Africa (75%) and the eastern Mediterranean region (62%). Africa achieved the largest total reduction, contributing 72% of the global reduction in measles mortality.[3] Measles vaccination coverage among infants in Southeast Asia and Africa is still low, ranging between 54%-55% in 1999 and 65%-67% in 2003. [4] However, Sri Lanka, Latin America,[5] Romania[6] and South Korea[7] experienced outbreaks of measles in spite of sustained high coverage with single-dose vaccination strategy. In India, measles was the major cause of mortality and morbidity in the pre-vaccination era. Measles immunization coverage in India increasing from 42.2%[8] to 50.7%[9] to 58.8%[10] suggests that there is gradual rise over the years; while in Himachal Pradesh, it is high coverage to 72%[11] to 89%[12] to 86%,[13] respectively. But in the country, Delhi state has higher vaccine coverage since it has taken the lead and initiative for the two-dose schedule of measles (first dose); and measles, mumps, rubella (MMR, second dose) at 9 months and 15 months, respectively. Goa, Maharashtra and Tamil Nadu reached 84%-88% coverage. The six states, viz., Andhra Pradesh, Chhattisgarh, Delhi, Gujarat, Punjab and Madhya Pradesh, achieved coverage of more than 70%.[14]

Population movement during the local festivals and the schools in the hilly areas facilitate transmission of the measles virus. Thus, outbreaks of measles are common among school children.[15,16] WHO and UNICEF recommend vaccinating all children from six months to 14 years of age along with vitamin A supplementation during emergencies.[17] In the month of September 2006, we investigated the double sequential outbreaks of measles under two sub-centers, viz., Sailli and Sarah, of Shahpur block of district Kangra. On the 14^th^ September 2006, a local community leader reported an outbreak of measles in the remote villages of Sailli, Kanol, Kutharna and Nauli under sub-center Sailli, with a population of 846, of Shahpur block. The villages are located in the remotest mountainous area of the district at an altitude of over 5000 feet. On the other hand, on the 26^th^ September 2006, a local health worker of sub-center Sarah reported an increase in the number of cases with febrile rash with white buccal spots (Koplik's spots) in the remote hilly villages of Jathrear, Bathrear and Gujrear, under sub-center Sarah, with a population of 430, of Shahpur block. The villages are located in the remote hilly area of the district at an altitude of over 3000 feet. We investigated the first outbreak on 21^st^ September 2006 followed by the second sequential outbreak on 28^th^ September 2006 with the objectives of confirming the diagnosis and formulating recommendations for prevention and control.

## MATERIALS AND METHODS

### Descriptive epidemiology

We defined a case clinically by WHO criteria as the occurrence of a febrile rash with or without cough, coryza and conjunctivitis in a resident of the seven villages of Shahpur block in the period from 1^st^ September to 30^th^ November 2006. Laboratory criteria employed for diagnosis was “at least a fourfold increase in antibody titer or isolation of measles virus or presence of measles-specific IgM antibodies.” Case classification includes clinically confirmed case, a case that meets the clinical case definition; probable is not applicable; and laboratory confirmed case indicates the case of a patient that meets the clinical definition and that is laboratory-confirmed, or linked epidemiologically to a laboratory-confirmed case.[18] Complications and deaths due to measles were considered if these occurred within 30 days of onset. We initiated active case search by visiting house-to-house to identify the cases that met the case definition or by stimulated passive surveillance in the aforementioned seven affected villages with a total population of 1276. The mother of every case patient or the next available elder member of the family was interviewed for 20 minutes using the semi-structured questionnaire in Hindi. The whole team was trained and supervised by two senior medical officers.

We line listed the case patients and out of them, those meeting the case definition were abstracted and described in terms of person, place and time characteristics. We also collected information about age; sex; place of residence; symptomatology and date of onset of illness; treatment taken, like modern/conventional medicine; immunization status of case patients, the susceptible population and assessment of cold chain system. We mapped the villages by location of households to show the distribution of the cases by residence. We calculated the attack rate of cases by age group, sex groups using population data obtained from the district health authorities. We examined the dynamics of the outbreaks by constructing an epidemic curve. We reviewed the use of vitamin A for case management during the outbreaks.

We collected administrative estimates of measles vaccine coverage. We also estimated the vaccine coverage in the population using the data gathered from mothers' interviews, immunization cards reviews and health care facility records reviews during field visit. We adopted retrospective cohort design to estimate the vaccine efficacy. We selected as study population the affected patients that were in the age groups from 10 months to 17 years. We calculated the preventable fraction of children among the exposed (i.e., those vaccinated) to obtain the vaccine efficacy. We used the following formula for cohort study: (attack rate among non vaccinated - attack rate among vaccinated) / attack rate among non vaccinated (The formula used was ARU–ARV/ARU*100; Table 1.)

**Table 1:** Attack rates of measles by age and vaccination status in seven villages — Shahpur block, district Kangra, Himachal Pradesh, India, in 2006

Name of the village	Age group in years	Children immunized against measles			Children not immunized against measles		
	
		Cases	Total	Attack rate %	Cases	Total	Attack rate %
Sailli	0-5	0	88	0	0	0	0
Kanol	0-5	0	116	0	0	0	0
Kutharna	0-5	0	91	0	0	0	0
Nauli	0-5	0	32	0	0	0	0
Total	0-5	0	327	0	0	0	0
Sailli	6-15	3	123	2.4	6	16	38
Kanol	6-15	3	169	2	2	15	13
Kutharna	6-15	27	129	21	4	15	27
Nauli	6-15	2	42	5	4	10	40
Total	6-15	35	463	7.5	16	56	29.5
Grand Total	0-15	35	790	4.4	16	56	28.6
Jathrear	0-5	0	80	0	0	0	0
Bathrear	0-5	0	63	0	0	0	0
Gujrear	0-5	0	32	0	0	0	0
Jathrear	6-10	4	40	10.0	1	3	33.33
Bathrear	6-10	1	54	1.90	0	2	0
Gujrear	6-10	1	41	2.40	0	3	0
Jathrear	11-17	6	45	13.33	2	7	29
Bathrear	11-17	1	26	3.84	1	4	25
Gujrear	11-17	1	27	3.70	0	3	0

(Relative risk: 6.44%; 95% confidence interval, 3.8-10.91; *P* <.001); (Proportion of the children vaccinated: 93.3%, with the calculated vaccine efficacy: 84.4%) under sub centre Sailli; (Relative risk: 5.3%; 95% confidence interval, 1.9-14.77; *P* <.001); (Proportion of the children vaccinated: 94.9%, with the calculated vaccine efficacy: 81.13%) under sub center Sarah

We explained the purpose of collecting the samples and the processing of the samples to the population of study areas. We took their written informed consent. In the fourth week of the ongoing outbreak, we collected (i) 16 random samples of 5 mL of blood for each specimen from different places, observing universal safety precautions (in total, 12 unpaired blood samples in the fourth week followed by four paired samples in the seventh week), and crystallized, separated the sera and refrigerated under +4°C to +8°C for testing specimens for IgM/IgG antibodies using ELISA; (ii) 5 samples of nasopharyngeal swabs in virus transport media (VTM) for virus isolation and genotyping of the strain by polymerase chain reaction; and (iii) 8 samples of 50 mL each of urine for culture/sensitivity using sterile equipment in virus transport media and all these samples were stored. We assigned international identification numbers and labeled other epidemiological details on all the samples. We transported the specimen to National Institute of Virology (NIV), Pune; and National Institute of Communicable Diseases (NICD), New Delhi, in reverse cold chain separately. The samples were only taken from those who were willing, while ten reluctant/refusing populations were dropped.

However, this investigation was conducted in the context of a public health response to an outbreak, and therefore ethical committee review was not indicated. We entered and analyzed the data by MS-Excel sheet and using Epi info version 3.3.2.

## RESULTS

**Figure 1:**
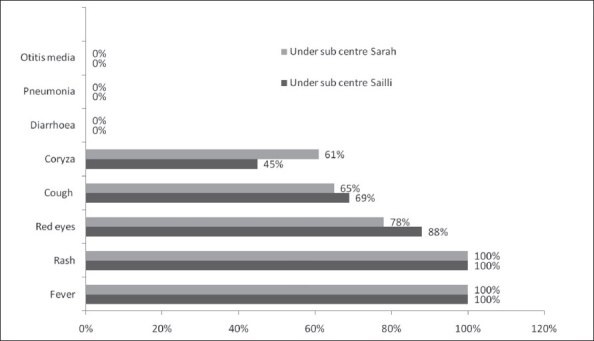
Symptomatology of case patients under sub-cente Sailli (*n* = 51) and sub-center Sarah (*n*=18) in double measles outbreaks of Kangra district, Himachal Pradesh, India, in 2006

In our study, we identified a total of 69 confirmed cases of measles (53/69 clinically confirmed and 16/69 laboratory-confirmed case patients from the study population). All 69 case patients belonged to the age group of 6 years-17 years. The attack rate (AR) among children 10 months to 17 years in the villages under both sub-centers ranged in between 1.7% and 25%, with the overall attack rate being 6% and the sex-wise attack rate being 8% in the sub-center Sailli [Table 2]. Median age of the case patients was 9 years (range, 6-15 years) under the Sailli sub-center, whereas it was 11 years (range, 6 years-17 years) under the Sarah sub-center. The area has a history of measles outbreak eight to ten years ago. The history of febrile rash was 100% in the all case patients [Figure 1]. The severity of the symptoms of the outbreak was less among the younger case patients and more (43/69) among the older ones, particularly in the lower socioeconomic strata, especially among illiterates as compared to literates (*P* >.00) and among scheduled castes (62%) as compared to others (*P* <.00). According to their mothers' statements, out of the 51 case patients in the four villages, 35 (69%) case patients were immunized. Block health administration conducted supplemental measles immunization in the form of providing ring immunization to the children in the four geographically difficult affected villages and in the adjoining villages. Out of the 18 patients in the three villages, viz., Jathrear, Bathrear and Gujrear, 14 (78%) patients were immunized.

**Table 2:** Age and sex-specific attack rates of measles in case patients in seven villages of Shahpur block of district Kangra, Himachal Pradesh, India, in 2006

Age group in years	Under sub-center Sailli								Under sub-center Sarah					
		
	Sailli		Kanol		Kutharna		Nauli		Jathrear		Bathrear		Gujrear	
							
	Cases/Total	Attack rate%	Cases/Total	Attack rate%	Cases/Total	Attack rate%	Cases/Total	Attack rate%	Cases/Total	Attack rate%	Cases/Total	Attack rate%	Cases/Total	Attack rate%
0–5	0/88	0.0	0/116	0.0	0/91	0.0	0/32	0.0	0/80	0.0	0/63	0.0	0/32	0.0
6-10	6/95	6.3	3/105	2.8	26/120	21.6	3/40	7.5	4/43	9.3	1/56	1.78	1/44	2.2
11-17	3/44	6.8	2/79	2.5	5/24	20.8	3/12	25	9/52	17.3	2/30	6.7	1/30	3.33
Sex-specific attack rate														
Male	33/412	8.0							10/243	4.1				
Female	13/434	3.0							8/187	4.3				
Total	51/846	6.0							22/430	4.2				

Out of the 69 patients, 51/69, (74%) case patients were administered vitamin A. Neither supplemental ring immunization nor vitamin A was provided to the children under sub-center Sarah. During the vaccination sessions, the cold chain was observed to be maintained properly. The temperature log book was also irregularly maintained at the sub-center Sailli. Out of the 69 patients, 15 (21%) opted for the traditional treatment of Vannan bushes (medicinal herbal plant) for three to five days as to and fro movement upon the chest and face of the case patients by the traditional healers (traditional healers *vs.* modern medicine, *P* <.049), and diet rich in seul, more so in Kutharna and Nauli areas (restricted diet *vs.* nutritious diet, *P* <.005); while 23 (33%) chose the modern system of medicine. Still, the majority of the case patients, i.e., 32 (46%) of the 69 patients, opted for both the treatment modes - traditional conservative one, first; and then, later on, switched over to modern medicine.

**Figure 2:**
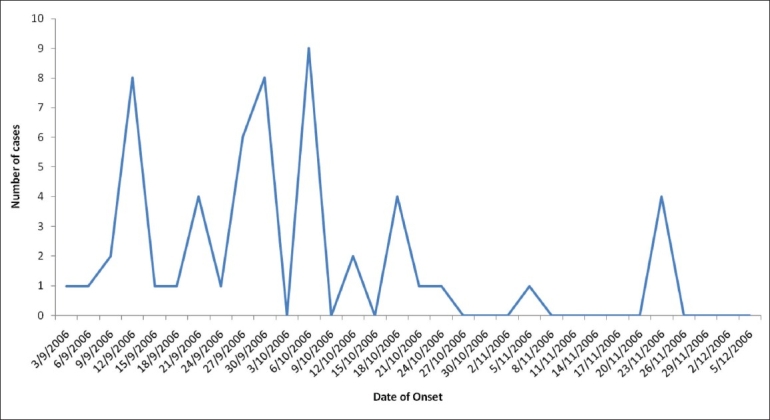
Epidemic curve by date of onset under sub-center Sailli (*n* = 51) and sub-center Sarah (*n* = 18) in double measles outbreaks of Kangra district, Himachal Pradesh, India, in 2006

There was sporadic distribution of the case patients in the households, with maximum number of case patients, 31/69 (44%), observed in Kutharna. The Kutharna village had three houses with more than 2 patients; while the village Kanol had one house with more than 1 case patient. In the first outbreak, the index case was identified in the area, reported on 1^st^ September 2006 from Kutharna village; and the maximum number of cases patients (9/31) was reported on 12^th^ September 2006, one incubation period after the inter-school game competition at Harchakiyan village. The second outbreak got the index case from Jathrear village due to cross-spread of infection from the local festival *Sayar* in Kutharna village. The dynamics of the two outbreaks in epidemic curve [Figure 2] indicated that there were a number of generations of cases, with the propagated outbreak peaking around 6^th^ October 2006. The number of cases declined during the third week of November 2006, and the outbreak ceased in the last week of November, with a fortnight free of any new case patients up to the second week of December 2006.

The administrative immunization coverage between 2001 and 2006 for children under the age of 5 years is listed in Table 3, whereas as per mothers' interview (the least specific criteria), it was 93% and 93%, in the Sailli and Sarah sub centers respectively while according to vaccination cards (the most specific criteria), it was 33% and 68% respectively.

**Table 3:** Reported measles vaccination coverage for the last five years (2001-2005) in sub-centers Sailli, Sarah and Shahpur block and subsequent theoretical calculation of the total number of susceptible individuals accumulated in the Shahpur block of district Kangra, Himachal Pradesh, India

Year	Birth cohort	Vaccination coverage of Shahpur block (%)	Vaccination coverage of sub-center Sailli covering affected 4 villages (%)	Vaccination coverage of sub-center Sarah covering affected 3 villages (%)	Expected V.E. (%)	Individual immunized each year in block	Susceptible individuals left each year in block	Cumulated number of susceptible individuals in block
2001	2763	104.2	106	99	85	2442	321	321
2002	2766	104.4	83	100	85	2445	321	642
2003	2748	107.8	78	98	85	2499	249	891
2004	2681	107.2	106	96	85	2438	243	1134
2005	2606	113.3	127	80	85	2503	103	1237

### Vaccine efficacy using analytical epidemiology

Attack rates of measles by age and vaccination status indicated that there were 16 case patients among the 56 (28.6%) non immunized children compared to 35 (4.4%) case patients among the 790 immunized children under sub-center Sailli; while in Sarah, ARs of measles by age and vaccination status indicated that there were 4 (18.18%) case patients among the 22 non immunized children compared to 14 (3.43%) case patients among the 408 immunized children; and both were statistically significant (*P* <.001) [Table 1]

Serologically, (i) 7/16 samples for measles IgM antibodies coupled with 4/16 paired samples with fourfold rise of IgG antibodies and (ii) 2/5 nasopharyngeal (NP) swabs for polymerase chain reaction test were positive; whereas (iii) all the eight urine samples, three nasopharyngeal samples and three blood samples leaked out during transportation due to mismanagement and hence no result was available for them. Measles D4 strain was genotyped in the two NP swabs, suggesting that D4 virus strains are circulating in district Kangra of Himachal Pradesh.

## DISCUSSION

Double sequential outbreaks of measles struck in seven remote villages under two sub-centers Sailli and Sarah in the months of September to November 2006. The first outbreak was first reported by the local pradhan of Kutharna panchayat and was not detected by the existing surveillance system, while the second outbreak was detected by the local health worker. The second outbreak got the index case from Jathrear village due to cross-spread of infection from local festival Sayar in Kutharna village, approximately 45 km climb-down from the sub-center Sailli (first outbreak). Although the patients during the outbreaks belonged to the lower socioeconomic strata and most of the patients were malnourished, yet the duration of illness and severity of the symptoms were less, particularly in the younger patients, but were severer in older patients. The serology proved IgM/IgG positive for measles, and D4 measles strain was genotyped. Symptoms frequency also supported the laboratory diagnosis. There was obvious age-shift towards the higher age group (6-17 years), maximally to the adolescent age group (11-17 years).[19] This suggests waning of immunity with age, which can be due to the use of poorly stored vaccine at the place of these children's vaccination. However, waning of immunity as an effect of exposure to ultraviolet radiation, as observed by Prof. Mary Norval, cannot be ruled out.[20] Low attack rates, no mortality and fewer complications reflected the mild nature of the outbreak, which in turn was because of better awareness and availability of health services. Despite the high coverage (>95%) in Shahpur block and in the affected villages under sub-centers Sailli and Sarah, inter-epidemic interval was more and the number of cases was sporadic,[21] thereby warranting the second dose of measles.[6,7] The administrative vaccination coverage has surpassed 100% due to placement of the additional camps in the left-out and the remote areas. The present double outbreak is due to the progressive accumulation of a small number of susceptible children in the community over the years [Table 3]. Such accumulations are typically caused by the combination of the following two factors: (1) the measles vaccine efficacy not reaching the level of 100% and (2) children being left un-immunized each year.

In the present study, many statistically significant factors are found to be associated with measles outbreaks, such as geographically difficult hilly areas, poor socioeconomic strata; illiteracy, marginalized sections like scheduled castes/tribes, overcrowding, traditional beliefs of people inducing them to seek help from the local chelas/quacks, program-related issues like irregular cold chain maintenance, etc. Similar findings have also been reported by other workers. [22–27]

Timely supplementary immunization activities and vitamin A supplementation were only implemented in the first outbreak; but in the second one, they were absent due to their non-availability at the headquarters at Shahpur for the last six months. Vitamin A supplementation has the protective role in reducing the morbidity and mortality during the measles outbreaks.[28] The cold chain was observed to be maintained properly all year round. However, there were few shortcomings, like improperly maintained temperature log book at the primary health center, Darini. There is a possibility of the failure of the vaccine potency as a result of irregular cold chain maintenance. A similar result has been seen in a highly vaccinated population by Lamb in 1989.[29]

Retrospective cohort study conducted during these outbreaks in the different villages generated evidence sufficient to suggest that the efficacies of the vaccines (84.4% and 81% in both sub-centers) were lower than anticipated, thereby warranting the requirement of the second dose opportunity for measles, to develop herd immunity.[30] Health systems and the millennium development goals use administrative measles vaccine coverage as a performance and development indicator. However, this indicator suffers from two limitations. First, it may overestimate the overall coverage. In the affected villages of district Kangra, although the low proportion of parents retaining the card of their child constituted a limitation of coverage surveys, yet in the villages, it was found that the coverage according to surveys was lower than the administrative estimates. Second, the overall estimate may not reflect pockets of lower immunization coverage. At present, there is no fixed rapid-response team in action. There was lack of trained persons in specimen collection and transportation. Logistics for specimen collection was not available.

### Limitation

(1) Recall bias could have occurred with respect to recollection of the details of immunization of the children of the areas. (2) Sero-surveillance in the study area could not be carried out due to constraints of funds and time.

## CONCLUSION

1. Double measles outbreaks were confirmed clinically, epidemiologically and serologically in highly immunized areas.
2. Vitamin A supplementation and Supplementary Immunization Activities were non-available in the second outbreak.
3. Surveillance system in place was weak in the first mountainous measles outbreak.
4. Traditional beliefs and barriers, mainly in marginalized families, formed the basis of treatment.
5. Defective practices of the cold chain system could have affected the effectiveness of the vaccine.
6. Medical human resource available was untrained, with inadequate logistics support and supply.

## RECOMMENDATIONS

1. We need to go for the second dose opportunity for measles in highly immunized areas/supplementary immunization activities (SIA) in left out difficult areas, with supplementation of vitamin A for case patients.
2. Measles surveillance needs to be strengthened through the upcoming Integrated Disease Surveillance Programme (IDSP) with the involvement of the private sector, particularly in urban areas.
3. Information, education and communication (IEC)activities should be addressed towards modifying the help-seeking behavior of mothers in the district, especially in the measles-affected areas.
4. Refresher training programs should be implemented for the workers of the affected areas for proper cold chain maintenance.
5. Availability of the logistics for specimen collection and transportation should be ensured.
